# Presentation of two patients with malignant granulosa cell tumors, with a review of the literature

**DOI:** 10.1186/1477-7819-10-185

**Published:** 2012-09-11

**Authors:** Ryousuke Tamura, Yoshihito Yokoyama, Takeshi Yanagita, Yukiko Matsumura, Kazuhiro Abe, Ryousuke Taniguchi, Hideki Mizunuma

**Affiliations:** 1 Department of Obstetrics and Gynecology, Hirosaki University Graduate School of Medicine, 5-Zaifu-cho, Hirosaki, Japan

**Keywords:** Ovarian malignant granulosa cell tumor, BEP combination therapy, Aromatase inhibitor

## Abstract

Granulosa cell tumors (GCTs) of the ovary account for 2 to 5 of ovarian malignancies. We present two patients with malignant ovarian adult GCT. In one patient, a combination of bleomycin, etoposide, and cisplatin was effective after initial surgery for malignant GCT. In the other, an aromatase inhibitor was effective for recurrent malignant GCT. We also review the literature for further management of this tumor. Because GCT of the ovary is rare, it will be necessary to elucidate the clinical phenotype and establish treatment protocols by accumulating and analyzing more patients.

## Background

Granulosa cell tumors (GCTs) of the ovary comprise 2 to 5% of malignant ovarian tumors [1,2], and 20 to 30% show malignant clinical and histopathological characteristics. The clinical outcome for tumors at stages I and II is excellent, whereas those at stages III and IV remains poor [3]. The 5-year survival rate for GCTs is approximately 80% overall, and some patients experience relapse 20 to 30 years after the initial surgery [4,5]. There are few reports on the clinical phenotype of malignant progression [6], and no standard therapy has yet been established [7]. We report our experience of two patients with malignant GCT, and review the literature.

## Case reports

### Patient 1

A 26-year-old woman presented to her doctor in December 2010 with abdominal pain and bloating. She had never been pregnant, but had a history of surgery for patent ductus arteriosus (at the age of 1 year) and also had type 2 diabetes. A pelvic neoplasm was suspected, and the patient was referred our department in January 2011 for detailed investigation and treatment.

Abdominal ultrasonography showed a mass, with hyperechogenicity. On magnetic resonance imaging (MRI) scan, an irregular, solid mass, 130 mm in size, was seen in the peritoneal cavity with associated pelvic ascites. Computed tomography (CT) showed enlargement of the left subclavian, para-aortic, and pelvic lymph nodes, and metastasis was thus suspected. No abnormality was seen with peripheral blood tests and biochemical tests, but the level of a tumor marker, CA125, was raised (233 U/ml; normal range 0 to 35 U/ml).

A diagnosis of malignant ovarian tumor was made, and surgery was performed. The laparoscopic findings indicated that the mass was an irregular, solid tumor derived from the left ovary. Because multiple distant metastases were present, a left adnexectomy was performed.

**Figure 1:**
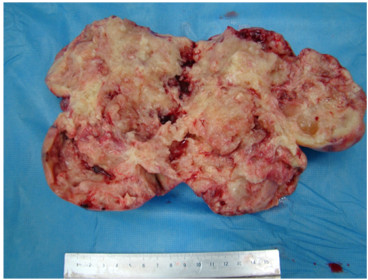
**Patient 1: macroscopic appearance of the tumor.** The cut surface of the tumor was yellowish and friable.

**Figure 2:**
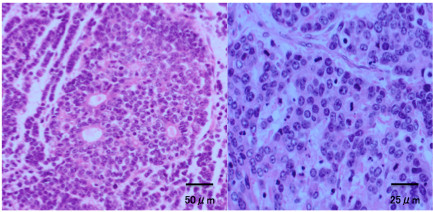
**Patient 1: histological appearance of the tumor.** Atypical cells were seen proliferating in a cord-like or alveolar configuration, and Call-Exner bodies were seen in some areas. Ten or more mitoses were present per high power field.

The cut surface of the tumor was yellowish and friable (Figure 1). On pathological examination, oval, atypical cells were seen, proliferating in a cord-like or alveolar configuration, and Call-Exner bodies were visible in some areas. Immunohistochemical staining gave positive results for alpha-inhibin. Under microscopic examination, 10 or more mitoses were present per high power field (Figure 2).

Based on these results, a malignant GCT of the ovary (Fédération Internationale de Gynécologie et d'Obstétrique (FIGO) stage IV) was diagnosed. After surgery, the patient was given three cycles of chemotherapy (BEP: bleomycin 20 mg/m^2^ on days 2, 9, and 16; etoposide 100 mg/m^2^ on days 1 to 3; cisplatin 15 mg/m^2^ on days 1 to 5) every 3 weeks.

A CT scan performed after the completion of two cycles showed a reduction in the size of lesions in lymph nodes at all sites. However, pyrexia and respiratory discomfort occurred, and worsened after the completion of three cycles. The patient was treated for a provisional diagnosis of drug-induced interstitial pneumonia, but her respiratory condition failed to improve, and she died as a result of respiratory failure caused by interstitial pneumonia.

### Patient 2

A 59-year-old woman was diagnosed with an ovarian tumor in December 1998, for which she underwent laparotomy. She had been pregnant four times and had three children; she had no other medical history of note.

**Figure 3:**
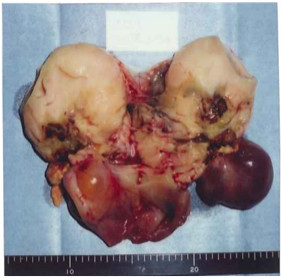
**Patient 2: macroscopic appearance of the initial tumor.** The cut surface was pale-yellow and mostly solid, with cystic areas containing mucus visible in some parts.

An intraoperative rapid pathological diagnosis of GCT was made, and a simple total hysterectomy, bilateral adnexectomy, omentectomy, pelvic lymphadenectomy, and para-aortic lymphadenectomy were performed. The tumor was an irregular mass derived from the left ovary. Although the cut surface was pale-yellow and mostly solid, cystic areas containing mucus were visible in some parts (Figure 3). The tumor was assessed as FIGO stage Ic. Adjuvant therapy was not given.

**Figure 4:**
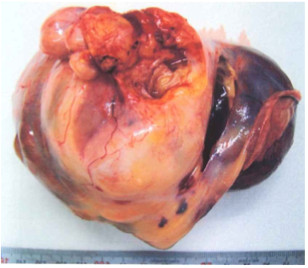
**Patient 2: macroscopic appearance of the recurrent tumor.** As with the initial tumor, the cut surface was pale-yellow and solid, with cystic areas containing mucus seen in some parts.

An abdominal CT performed in February 2004 showed an intraperitoneal mass, indicating tumor recurrence. The patient was given 10 cycles of chemotherapy (IEP: ifosfamide 800 mg/m^2^ on days 1 to 3; epirubicin 50 mg/m^2^ on day 1; ciclosporin 15 mg/m^2^ on days 1 to 5), with one cycle every 3 weeks, which resulted in a reduction in the size of the lesion. However, re-expansion of the deposits occurred, and total resection of the intraperitoneal recurrences was attempted in October 2006 (Figure 4). All recurrences presented as multiple intraperitoneal masses, ranging in size from 50 to 100 mm. The cut surface of the tumors was pale yellow and solid, and cystic areas containing mucus were seen in some areas (Figure 4), indicating macroscopic findings similar to those at the time of initial surgery. Based on the post-operative pathological examination, a malignant GCT was diagnosed, and the patient was given six cycles of post-operative chemotherapy (TC: paclitaxel 180 mg/m^2^ on day 1; carboplatin (area under the curve 5) on day 1) every 3 weeks.

**Figure 5:**
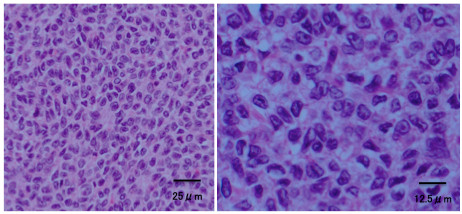
**Patient 2: histological appearance of the recurrent tumor.** Call-Exner bodies, coffee bean-like cells, and mitoses were visible.

A CT scan in April 2009 indicated an intraperitoneal mass, and a second resection of recurrent, intraperitoneal masses was performed in May 2009. Total resection was also attempted on this occasion. After surgery, the patient was treated with an oral aromatase inhibitor (anastrozole) 1 mg/day, but a CT scan in September 2010 showed recurrent intraperitoneal masses. Because enlargement was also seen, a third, complete resection was attempted in May 2011. Pathological examination showed atypical cells proliferating in a mixed solid and cystic manner in all specimens in the recurrences obtained in 1998, 2006, 2009, and 2011, and Call-Exner bodies, coffee bean-like cells, and mitoses were also visible (Figure 5). Immunohistochemistry gave a positive result for alpha-inhibin.

Based on the above results, a malignant GCT was diagnosed. Oral administration of anastrozole 1 mg/day has continued with the patient followed up as an outpatient, and this has resulting in disease-free survival to the most recent follow-up (April 2012).

## Discussion

Ovarian GCT is classified as a sex cord-stromal tumor and is treated as a borderline malignant tumor. These tumors show the clinical and pathological features of malignancy at a rate of 20 to 30%, and comprise 2 to 5% of ovarian malignant tumors [1,2]. Prognostically, the 5-year survival rate of patients with stage I or II disease is thought to be 95%, and that of patients with stage III or IV disease is 59% [8]. However, some patients experience relapse 20 to 30 years after the initial surgery [4,5], and there have been a few reports published on the clinical phenotype of those with rapid progression [6]. Jozwicki *et al*. reported a patient with a very aggressive ovarian GCT, composed of granulosa, sarcomatoid, and fibrothecomatous tissues [6]. The tumor recurred rapidly, and the patient died 16 months later [6].

The histopathological characteristics of GCTs include cells with a coffee bean-like longitudinal nuclear groove and a micro-follicular structure termed the ‘Call-Exner body’. In patients with malignancy, a large number of mitoses and dyskaryoses are also seen. According to one report, the number of mitoses is a pathological prognostic factor [9]. Alpha Inhibin-alpha is a sensitive immunohistochemical marker of GCT of the ovary, and is of value in the differential diagnosis of ovarian neoplasia [10] as shown in the two patients presented.

Because it is difficult to distinguish GCTs from epithelial ovarian tumors pre-operatively, and because the accuracy of intraoperative rapid pathological diagnosis is not good, basic techniques used for the management of ovarian cancer (bilateral adnexectomy plus+total hysterectomy+plus omentectomy) and staging laparotomy are recommended as the initial therapy [11]. Indications and protocols for adjuvant therapy have not yet been established because GCTs are rare, and a large-scale clinical study has not been conducted. In general, chemotherapy is recommended for patients with residual foci, a high risk of recurrence (tumor rupture, stage Ic or greater, a poorly differentiated tumor, or a tumor diameter of >100 to 150 mm); or actual recurrence. PVB (cisplatin + vinblastin + bleomycin) therapy [12] and BEP (bleomycin + etoposide + cisplatin) therapy [13] are considered effective regimens, based on the results of clinical studies that showed a relatively high response rate. The target of both therapies is the progressive, recurrent GCT, and the response rates to PVB and BEP therapies were given as 60.5% and 37%, respectively [12,13]. Although retrospective, a study of the combination therapy of taxane-based drugs and platinum-based drugs in patients with recurrences showed a response rate of 54% [14]. Drugs with an anti-estrogenic effect, such as gonadotropin-releasing hormone agonists and aromatase inhibitors, have recently been suggested to be effective [15-17]. Aromatase inhibitors exhibit an anti-estrogen effect by binding to aromatase, an enzy\me required for the conversion of androgen to estrogen, and inhibiting its activity. Although aromatase inhibitors are used clinically to treat post-menopausal women with estrogen receptor-positive breast cancer [18], there are some reports of patients in whom an aromatase inhibitor was effective against GCTs with repeated recurrences [15-17], as for patient 2.

Patient 1 had a reduction in the size of lesions in lymph nodes after BEP therapy, confirming the efficacy of the treatment. However, because serious interstitial pneumonia also occurred in this patient, as has been reported for other cases in which bleomycin was implicated in treatment-related death, the use of this treatment needs to be reconsidered. By contrast, even though the intraperitoneal tumor recurred in patient 2 after the start of aromatase inhibitor administration, the tumors were small, suggesting that the aromatase inhibitor slowed tumor growth, and that this drug is effective against recurrent tumors.

## Conclusions

The results for our two patients suggest that BEP therapy is effective after initial surgery and that an aromatase inhibitor is effective for recurrent tumors. However, because GCTs are rare, it will be necessary to elucidate the clinical phenotype and establish treatment protocols by accumulating and analyzing more patients.

## Consent

Written informed consent was obtained from the patient or the patient’s family for publication of this case report and any accompanying images. A copy of the written consent is available for review by the Editor-in-Chief of this journal.

## Competing interests

The authors declare that they have no competing interest.

## Authors’ contributions

RT, YY, TY, YM, KA, RT and HM treated the two patients as a team and reviewed the literature. RT drafted the manuscript, and YY, TY, YM, KA, RT and HM also helped to draft the manuscript. All authors read and approved the final manuscript.

## Authors’ information

RT, TY, YM, KA and RT work as instructors at Department of Obstetrics and Gynecology, Hirosaki University Graduate School of Medicine. YY is an associate professor and HM is a professor and is also chair of the Department of Obstetrics and Gynecology, Hirosaki University Graduate School of Medicine.
